# PIOS (Patras Immunotherapy Score) Score Is Associated with Best Overall Response, Progression-Free Survival, and Post-Immunotherapy Overall Survival in Patients with Advanced Non-Small-Cell Lung Cancer (NSCLC) Treated with Anti-Program Cell Death-1 (PD-1) Inhibitors

**DOI:** 10.3390/cancers12051257

**Published:** 2020-05-16

**Authors:** Foteinos-Ioannis Dimitrakopoulos, Achilleas Nikolakopoulos, Anastasia Kottorou, Fotini Kalofonou, Elias Liolis, Theodora Frantzi, Ioannis Pyrousis, Angelos Koutras, Thomas Makatsoris, Haralabos Kalofonos

**Affiliations:** 1 Division of Oncology, Department of Medicine, University Hospital of Patras, 26504 Rion, Greece; fodimitrakopoulos@upatras.gr (F.-I.D.); achylles@otenet.gr (A.N.); lioliselias@yahoo.gr (E.L.); theodorafrantzi@gmail.com (T.F.); angkoutr@otenet.gr (A.K.); maktom@yahoo.com (T.M.); 2 Clinical and Molecular Oncology Laboratory, Medical School, University of Patras, 26504 Rion, Greece; agathonisiotisa@yahoo.gr (A.K.); ioannis.pyrousis@upnet.gr (I.P.); 3 Imperial Healthcare Trust-Oncology, London W2 1NY, UK; kalfotini@gmail.com

**Keywords:** NSCLC, immunotherapy, lung cancer, prognostic, predictive, biomarkers, nivolumab, pembrolizumab, Patras Immunotherapy Score, PIOS

## Abstract

Immunotherapy with immune checkpoint inhibitors (ICIs) has changed the therapeutic management of advanced non-small cell lung cancer (aNSCLC) over the last decade. However, there is an unmet need for clinically useful biomarkers in this patient subgroup. The aim of this study was to combine baseline clinical characteristics of aNSCLC patients, in the form of a scoring system, and to investigate its predictive and prognostic value in NSCLC patients treated with ICIs. A total of 112 patients with advanced (stages IIIA to IV) NSCLC, treated with nivolumab or pembrolizumab, were enrolled in this study. Patras Immunotherapy Score (PIOS) was developed based on four of the studied parameters (performance status (PS), body mass index (BMI), age, and lines of treatment (LOT), which were incorporated into our formula (PS × BMI/ LOT × age). PIOS score was strongly associated with best overall responses (BOR), with those patients having benefit/good response (stable disease (SD) or partial (PR) or complete response (CR), achieving a higher score compared to patients who developed progressive disease (PD) (*p* < 0.001). Furthermore, PIOS score was associated with progression-free survival (PFS), since high-score patients had longer PFS (*p* < 0.001, hazard ratio (HR) = 0.469). Moreover, PIOS was associated with post-immunotherapy overall survival (OS), with high-score patients having improved OS (log-rank *p* = 0.019). This study suggests that a combination of baseline parameters, which give rise to PIOS score, may predict the best response of NSCLC patients treated with anti-program cell death -1 (PD-1) monotherapy as well as it may have a potent prognostic value for PFS and post immunotherapy OS.

## 1. Introduction

Immunotherapy is a major breakthrough in the treatment of advanced non-small-cell lung cancer (aNSCLC) during the last decades, changing the landscape of medical management of this cancer type [1]. Immunotherapy has not only changed the treatment options as second line and beyond, but has also evidence as first line treatment of aNSCLC [2]. Immune checkpoint inhibitors (ICIs), targeting programmed-cell death 1 (PD-1) and programmed-cell death ligand 1 (PD-L1) are the first-in-class immunotherapeutic drugs approved for management of aNSCLC, with the existing data supporting their use in earlier stages of the disease as well, in the near future [3]. ICIs have a great clinical impact in NSCLC, as the role of these monoclonal antibodies lies on the reactivation of the immune response, through targeting and interrupting the signaling via the PD-1/PD-L1 axis [4]. 

Nivolumab, was the first-in-class ICI, which was initially approved in early 2015 by the US Food and Drug Administration (FDA), as a second line of treatment in advanced squamous non–small-cell lung cancer (NSCLC), after progression with platinum-based chemotherapy (CheckMate 017 study) [5]. Subsequent to this approval, nivolumab was also approved a few months later for the treatment of patients with metastatic non-squamous NSCLC, with progression on or after platinum-based chemotherapy, according to the results of CheckMate 057 trial [6]. Pembrolizumab, another monoclonal antibody against PD-1, has also been approved for the second-line treatment of NSCLC patients, with at least 1% PD-L1 expression, as well as for the first line treatment in cases with PD-L1 overexpression (over 50%), or in combination with chemotherapy, irrespective of the PD-L1 status [4]. 

Currently, PD-L1 expression and tumor mutation load (TML) or tumor mutation burden (TMB) are the two main independent biological traits of NSCLC, having been validated for their predictive value in terms of immunotherapy [7]. In particular, percentage of PD-L1 expression is the most extensively studied and validated marker, as demonstrated in a great number of clinical trials [8]. Additionally, Rizvi et al. have shown that higher nonsynonymous mutation burden in tumors is associated with improved objective response and progression-free survival, since higher TMB implies the generation of more neoantigens [9].

Despite the tremendous reform ICIs have brought into the treatment landscape, only a part of advanced stage NSCLC patients, treated with ICIs, do experience significant and durable response, as well as a survival benefit [7]. Unfortunately, with the exception of PDL-1 expression and TMB, no other validated predictive biomarkers exist to date [10]. Therefore, there is an unmet need for clinically useful predictive biomarkers for ICIs, in order to identify those NSCLC patients with the highest potential to achieve durable responses, as well as the ones due to develop serious ICIs related to adverse events [11].

In the current study, an effort was made to establish and evaluate a novel predictive score for NSCLC patients, with advanced disease, treated with anti-PD-1 monotherapy (nivolumab or pembrolizumab), while incorporating non-interventional, baseline, clinical parameters. 

## 2. Results

### 2.1. Patients’ Characteristics

Totally one hundred and twelve NSCLC patients were enrolled in the current study. Patients’ characteristics are summarized in Table 1. All patients were treated with anti-PD-1 monotherapy (nivolumab or pembrolizumab), in the context of the advanced disease. The majority of the participants were male (76.8%), while 23.2% of them were females. The median age was 67 years (range 39–84 years) with the vast majority of them (86.7%) being current or former smokers. Most of the recruited patients had a confirmed histological diagnosis of adenocarcinoma or squamous cell carcinoma; 14 patients with NSCLC were impossible to be further categorized. Only three of the patients had mutation of the EGFR and none of them bore ALK translocations, while 27 patients had PD-L1 expression over 1%. Nivolumab was exclusively used in 94 patients beyond first line, while pembrolizumab was administered in 13 patients as front-line treatment and in five patients in second line and beyond. All patients had advanced disease with stages 3B, 3C, or stage 4 disease, while two patients with stage 3A disease were also enrolled, as they had inoperable disease.

Performance status (PS) of the enrolled patients was evaluated using the median Eastern Cooperative Oncology Group performance status (ECOG PS), prior to and at the time of anti-PD-1 initiation. Most of the patients had PS 0 (48.2%), 42% had PS 1, and 11 patients had PS 2 or 3. Responses and survival outcomes were available for all cases included in the study. Data cutoff was performed in March 2020 and more than 60% of our cohort had achieved a good best overall response (BOR) to immunotherapy (stable disease (SD), partial response (PR), complete response (CR) at the time (62.5%). The remaining 42 patients had progressive disease (PD), based on the evaluation of disease stage from the first follow-up after the initiation of the immunotherapy. Almost half of the patients (53.6%) died during the observational period. 

### 2.2. PIOS Score Was Associated with Best Response to Anti-PD-1 Treatment

Available non-interventional clinical parameters and possible combinations were studied, with regard to their predictive value, on immunotherapy response. Four of the clinical parameters measured prior to initiation of ICIs, including PS, body mass index (BMI), age, and lines of treatment (LOT), were combined and incorporated into our formula (PS×BMI/LOT×age), giving rise to PIOS (Patras Immunotherapy Score). Furthermore, PIOS predictive value was evaluated in our patient cohort. PIOS was strongly correlated with BOR, with good responders (patients with SD, PR or CR) having higher scores compared to those with PD in a two-tier model (*p* < 0.001). The association was significant, even with the use of a four-tier model (PD, SD, PR, and CR) for BOR (*p* < 0.001). After Bonferroni adjustment for multiple tests, PIOS score differed between patients with PD and SD (*p* = 0.046) and between patients with PD and PR (*p* < 0.001), as well as between patients with PD and CR (*p* = 0.002). Predictive significance of PIOS score (median) also persisted using a binary logistic regression analysis, adjusted for age and histological subtype (*p* = 0.001, hazard ratio (HR) = 0.200, 95%, confidence interval (CI) 0.077–0.517). 

### 2.3. PIOS Was Associated with PFS and Clinical Outcome

**Figure 1:**
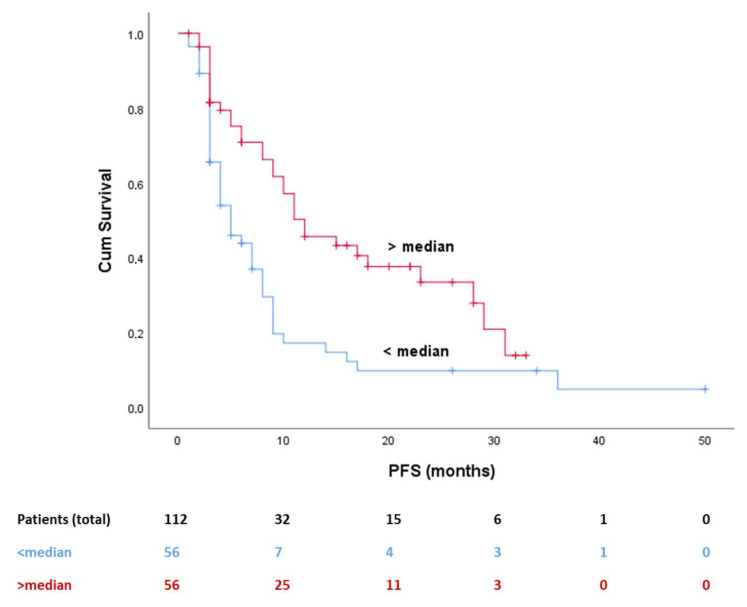
Kaplan–Meier curve for PFS and PIOS (median value used as cutoff point). Abbreviations: PFS, progression free survival; PIOS, Patras Immunotherapy Score.

PIOS was also associated with progression-free survival (PFS), since patients with higher PIOS score were related to longer PFS (Figure 1, log-rank *p* < 0.001). Median PFS was 15 months for the favorable subgroup and five months for the poor responders (HR 0.469, 95% CI 0.295–0.747). Multivariate analysis for PFS, adjusted for weight and PS, confirmed the prevalence of the predictive value of PIOS (Table 2, HR 0.023, 95% CI 0.001–0.590, *p* = 0.027).

### 2.4. PIOS Was Associated with Clinical Outcome

**Figure 2:**
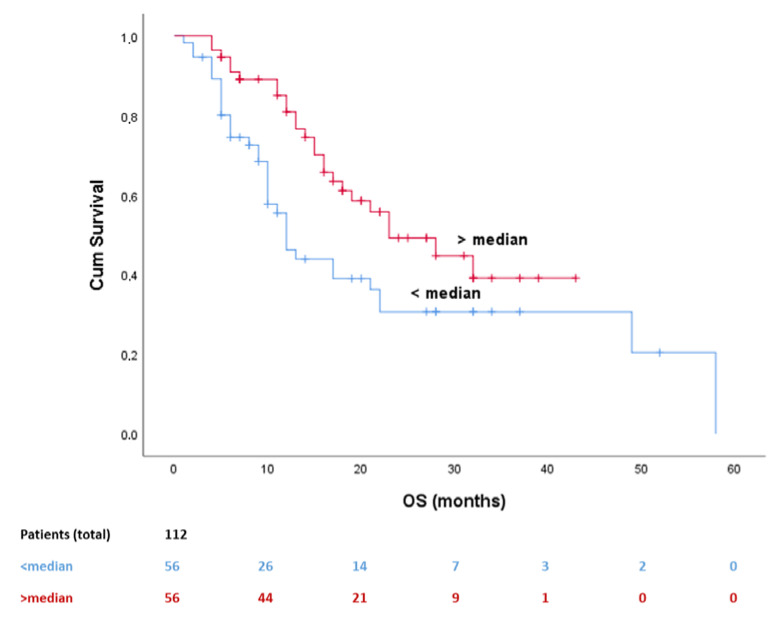
Kaplan–Meier curve for post immunotherapy OS dividing patients in two different predictive groups. OS, overall survival.

At univariate analysis, PIOS was also associated with post-immunotherapy overall survival (OS) with patients with higher PIOS score (over median) having improved OS (log-rank *p* = 0.019). Median OS was 32 months for the favorable subgroup and 14 months for the poor responders (Table 3, HR = 0.539, 95% CI 0.317–0.918). Potential covariates, sex (*p* = 0.049), histological subtype (*p* = 0.017), PS (*p* < 0.001), and LOT (*p* = 0.051) were counted for the multivariate analysis. After adjustment, PIOS score remained statistically significant (Table 2, *p* = 0.030, HR = 0.001, 95% CI 0.000–0.571) (Figure 2). 

### 2.5. PIOS Was Associated with TtBR, TiBR, and TTBR

**Figure 3:**
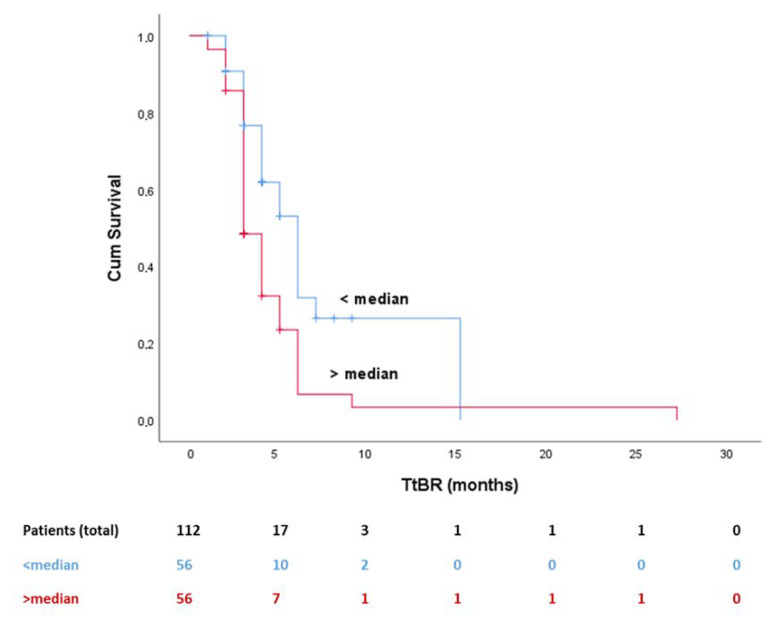
Kaplan–Meier curve for TtBR and PIOS (median value used as cutoff). Abbreviations: TtBR, time to best response; PIOS, Patras Immunotherapy Score.

In addition, based on time to event (BOR) analysis, PIOS was associated with time to best response (TtBR), since patients with higher (>median) scores achieved faster BOR compared to patients with lower scores (Figure 3, *p* = 0.001). Additionally, patients with higher PIOS score (>median) had longer time in best response (TiBR) (*p* = 0.017) and total time in best response (TTBR) (*p* = 0.028), experiencing, therefore, a longer survival benefit, compared to the ones with low PIOS score. 

## 3. Discussion

Over the last decade, a significant improvement has been achieved in the therapeutic options available for the treatment of NSCLC, especially with the introduction and broad use of ICIs, strongly incorporating immunotherapy in the armament of medical oncologists worldwide [4]. ICIs have changed the clinical course of patients with advanced NSCLC [12]. Although a substantial portion of patients do not experience a clinical benefit, a significant survival improvement for a part of them has been observed, not only in clinical trials, but also in real-world data [4]. In this vein, predictive biomarkers represent an urgent need, especially when we consider the rare but existing detrimental side effects as well as the financial toxicity of immunotherapy (IO) for modern societies [11].

In this study, we established and evaluated a new predictive score, PIOS, which is associated with the response to ICIs. The PIOS ratio can be calculated using four clinically useful and non-interventional simple parameters (PS, BMI, LOT, age). All of these parameters have separately been assessed and considered as predictive or prognostic factors. However, this is the first report to highlight the significance of the combination of all four of them as a new predictive scoring system. Prelaj et al. have reported that ECOG PS 2 have a negative predictive value in NSCLC [13]. In addition, Ahmed et al. have documented that although baseline PS did not demonstrate any correlation with response, poorer PS was associated with inferior PFS and OS [14]. Unfortunately, patients with poor PS are under-represented in clinical trials, since it has been hypothesized that a poor PS represents a deteriorated immune system with lymphocytes with decreased functionality [13].

BMI is another representative factor of the PIOS. The potent predictive value that BMI has in cancer immunotherapy is previously known in literature. Findings from an increasing number of studies have underlined a potential correlation between BMI and the efficacy of ICIs. A recent retrospective study by Cortellini et al. showed that in a panel of patients with NSCLC, melanoma, renal cell carcinoma, and other types of cancer, treated with ICIs, overweight or obese patients had higher response rates and longer survival outcomes compared to patients with lower BMI [15].

The other component of the PIOS formula, age, is a clinical parameter with a known potential to predict effectiveness of anti-PD-1 treatment. Kugel et al. reported that older melanoma patients are more likely to respond to immunotherapy and that each decade of life decreases the chance of disease progression after anti-PD-1 therapy [16]. On the other hand, no difference has been reported across age groups in melanoma patients, with regard to survival outcome [17]. Similarly, Marur et al. have documented that older NSCLC patients (≥65 years), with advanced or metastatic disease, share similar survival benefits from ICIs’ treatment, with younger patients [18]. In addition, Botticelli et al. have documented that elderly treated with nivolumab seem to have a survival advantage compared to younger patients, although the difference did not reach statistical significance (*p* = 0.057) [19]. 

PIOS score is also empowered with prognostic value, since it has been associated with PFS, as well as post-immunotherapy OS, in NSCLC patients treated with anti-PD-1 inhibitors. The principal interest of ongoing research regarding potent prognostic biomarkers for ICs has so far focused on evaluation of immune system-related features. Neutrophil-to-lymphocyte ratio (NLR), assessed in peripheral samples from different cancers, is one of the most evaluated biomarkers. Increased NLR scores have been related to shorter survival in NSCLC patients treated with ICIs [20], as well as with hyper-progressive disease [21]. The prognostic significance of NLR in patients treated with nivolumab has also been shown in a meta-analysis, which enrolled 14 retrospective studies [22]. The combination of immune related with clinical traits has been evaluated as well, such as the case of, ALI (advanced lung cancer inflammation index) [23], AISI (aggregate index of systemic inflammation) [24], SII (systemic inflammation index) [25], LIPI (lung immune prognostic index) [20], EPSILoN (ECOG PS, smoking history, evidence of liver metastases, levels of lactate dehydrogenase (LDH), and neutrophil-to-lymphocyte ratio (NLR)) [13] and iSEND (immunotherapy sex-ECOG-NLR-delta NLR) [26].

Despite our promising results, we have to acknowledge some limitations in the current study. A weakness of our study is the analysis in part of the retrospectively collected data, although almost half of the patients were prospectively collected. Another weakness is the number of patients. A larger cohort would be required to achieve more robust results. Moreover, molecular profile regarding EGFR mutations, ALK rearrangements and PD-L1 status were not available for all patients, due to inexistence of tissue samples or because of quality restrictions. Furthermore, although the current study was designed to follow a two-phase design, including a validation group, the final presentation of results is a pool analysis of the whole cohort.

Despite the aforementioned limitations, the major advantage of our study is the inclusion in the PIOS score of only baseline, clinical, and non-interventional parameters, which are routinely collected. Due to the incorporation of only these parameters, PIOS score has the potential to be put into everyday oncology practice.

## 4. Patients and Methods

### 4.1. Study Design, Population, and Data Collection

Helsinki Declaration on ethical guidelines was followed during the conduction of this study (2013) [27] and it was initially approved by the Scientific Committee and the Committee on Research and Ethics of the University Hospital of Patras (No 3/18-2-2015, Greece). 

Patients enrolled in the current study were retrospectively and prospectively recruited. Initially, clinical parameters were assessed in a retrospectively recruited group, followed by a second, prospectively recruited, patient cohort. Although our preliminary results from the retrospective group were validated in the prospective group, statistical analysis presented here was performed in the sum of the cases, for robustness in the statistical outcome. 

All the patients had histologically or cytologically confirmed diagnoses of NSCLC (adenocarcinomas or squamous carcinomas). The patients were medically managed with the anti-PD-1 ICIs nivolumab or pembrolizumab at the Division of Medical Oncology, Department of Medicine of the University of Patras, Greece, between the years 2015–2019, according to the treatment guidelines of the time. 

Patients enrolled in the current study were selected blindly to the response and the clinical outcome. Patients with follow-up data not available were excluded from our analysis. Incomplete administration of ICIs, use of additional anti-neoplastic drugs and administration of high dose of glucocorticoids were among the exclusion criteria. Collection data included clinicopathological traits, performance status (ECOG PS) before and after ICIs’ initiation, smoking history, molecular profiling for epidermal growth factor receptor (EGFR,) and anaplastic lymphoma kinase (ALK), previous treatments, number of ICIs cycles, TtBR, BOR based on the clinical evaluation or radiological reports, TiBR, TTBR, PFS, post-immunotherapy OS, and last follow-up or date of death. ECOG PS was converted to Karnofsky Performance Scale (Karnofsky PS) for calculation reasons, according to findings from Prasad et al. [28]. Additionally, immunotherapy-related side effects and toxicity data were also collected. PD-L1 and molecular status were available only if such profiling had been performed, as part of the routine clinical care. PD-L1 expression was assessed using the Dako immunohistochemical assay (Dako; Carpinteria, CA, USA) and scored according to tumor proportion score (TPS).

### 4.2. Statistical Analysis

Statistical analysis was performed using the Statistical Package for Social Sciences version 17 (SPSS, IBM Corp, Armonk, NY, USA). Frequencies and percentages were used for the description of categorical as well as medians and ranges for quantitative variables. Categorical nominal variables were evaluated using the Chi-square test or Fisher exact test. The t test was used for continuous variables with normal distribution. Analysis for ordinal or continuous data was performed by using Kruskal–Wallis or the Mann–Whitney tests. Additionally, in order to identify whether studied parameters and PIOS score were independently related to response, we used binary logistic regression models.

Survival analysis and plotting was performed using the Kaplan–Meier method and the log-rank test. Multivariate analysis of the studied molecules was assessed by Cox proportional hazards models, in order to clarify if the evaluated parameters were associated with TtBR, TiBR, TTBR, PFS, and OS. Median of PIOS score was used as cutoff point. For all comparison purposes, statistical significance was defined at 5% and all tests were two-sided.

## 5. Conclusions

In conclusion, this study shows that PIOS score, which is based on a combination of clinical and non-interventional parameters, seems to have the ability to predict the best response of NSCLC patients treated with anti-PD-1 monotherapy. Moreover, this score has prognostic value for PFS and post immunotherapy OS, offering substantial clinical information for NSCLC patients treated with ICIs.

## Figures and Tables

**Table 1:** Characteristics of NSCLC patients enrolled in the current study.

Patient Characteristics	Number (%)
**Total**	112
**Age (years) median (range)**	67 (39–84)
**BMI mean (range)**	26.08 (17.4–44.6)
**Gender**	
Male	86 (76.8)
Female	26 (23.2)
**Smoking status**	
Current	76 (67.9)
Former	21 (18.8)
Never	8 (7.1)
NA	7 (6.3)
**Pack-years mean (range)**	72.4 (0–200)
**Histology**	
Total	112
Squamous	45 (40.2)
Non-squamous	53 (47.3)
NSCLC	14 (12.5)
**Stage**	
IIIA	2 (1.8)
IIIB	31 (27.7)
IIIC	5 (4.5)
IV	74 (66.1)
**Primary location**	
Left lung	51 (45.5)
Right lung	57 (50.9)
NA	4 (3.6)
**Lymph node infiltration**	
No	20 (17.9)
Yes	88 (78.6)
NA	4 (3.6)
**Grade**	
I	4 (3.6)
II	19 (17.0)
III	57 (50.9)
NA	32 (28.6)
**Molecular status**	
EGFR mutations	3 (2.7)
ALK translocations	0 (0)
**PD-L1 status**	
Positive (>1%)	27 (24.1)
Negative	9 (8.0)
NA	76 (67.9)
**PS**	
0	54 (48.2)
1	47 (42)
2	8 (7.1)
3	3 (2.7)
NA	0 (0)
**Lines of treatment (LOT)**	
1	13 (11.6)
2	77 (68.8)
≥3	22 (19.6)
NA	0 (0)
**Regimen**	
Nivolumab	94 (83.9)
Pembrolizumab	18 (16.1)
**Best overall response (BOR)**	
CR	4 (3.6)
PR	40 (35.7)
SD	26 (23.2)
PD	42 (37.5)
NA	0 (0)
**Final outcome**	
PD	76 (67.9)
SD, PR, CR	19 (17.0)
NA	17 (15.2)
**Overall survival status**	
Alive	52 (46.4)
Dead	60 (53.6)

Abbreviations: BMI, body mass index; NA, not available; NSCLC, non-small-cell lung cancer; PS, performance status; CR, complete response, PR; partial response; SD, stable disease; PD, progressive disease.

**Table 2:** Univariate and multivariate analysis for PFS.

Covariate	Univariate Analysis		Multivariate Analysis	
	HR (95% CI)	*p* Value	HR (95% CI)	*p* Value
**Age ≥ 67 years**	1.386 (0.886–2.168)	0.131		
**Sex**	1.353 (0.796–2.297)	0.238		
**Weight ≥ 74.5 kg**	0.547 (0.348–0.859)	**0.005**	0.650 (0.402–1.049)	0.077
**Height ≥ 1.68 m**	1.157 (0.738–1.815)	0.502		
**BMI ≥ 26.26**	0.738 (0.471–1.156)	0.160		
**BSA ≥ 1.84**	0.723 (0.460–1.136)	0.137		
**Location (left)**	1.129 (0.721–1.767)	0.576		
**Histology (SQ)**	1.378 (0.860–2.207)	0.158		
**Stage (3)**	1.028 (0.648–1.631)	0.903		
**Infiltrated LN (No vs. Yes)**	1.364 (0.755–2.462)	0.276		
**Smoking (Current)**	0.859 (0.516–1.429)	0.535		
**PS (0 or 1)**	0.316 (0.160–0.625)	**<0.001**	0.367 (0.180–0.750)	**0.006**
**LOT (First)**	0.489 (0.212–1.126)	0.072		
**PIOS score ^+^**	0.469 (0.295–0.747)	**0.001**	0.023(0.001–0.590)	**0.023**

^+^ Univariate analysis was performed using median as cutoff. *p* values in bold represent statistically significant results. Abbreviations: PFS, progression free survival; BMI, body mass index; BSA, body surface area; SQ, squamous cell carcinoma; LN, lymph nodes; PS, performance status, LOT, lines of treatment, PIOS, Patras Immunotherapy Score.

**Table 3:** Univariate and multivariate analysis for OS.

Covariate	Univariate Analysis		Multivariate Analysis	
	HR (95% CI)	*p* Value	HR (95% CI)	*p* Value
**Age ≥ 67 years**	1.306 (0.777–2.195)	0.304		
**Sex**	1.944 (0.980–3.856)	0.049	1.794 (0.861–3.736)	0.119
**Weight ≥ 74.5 kg**	0.736 (0.438–1.237)	0.237		
**Height ≥ 1.68 m**	1.515 (0.895–2.565)	0.113		
**BMI ≥ 26.26**	0.853 (0.507–1.436)	0.542		
**BSA ≥ 1.84**	0.820 (0.487–1.383)	0.449		
**Location (left)**	0.855 (0.507–1.443)	0.551		
**Histology (SQ)**	1.925 (1.105–3.355)	**0.017**	2.417 (1.317–4.438)	**0.004**
**Stage (3)**	0.867 (0.511–1.470)	0.590		
**Infiltrated LN (No vs. Yes)**	1.117 (0.577–2.163)	0.743		
**Smoking (Current)**	0.792 (0.446–1.404)	0.415		
**PS (0 or 1)**	0.240 (0.115–0.499)	**<0.001**	0.199 (0.081–0.492)	**<0.001**
**LOT (First)**	0.337 (0.105–1.080)	0.051	2.533 (0.505–12.706)	0.259
**PIOS score ^+^**	0.539 (0.317–0.918)	**0.019**	0.001 (0.000–0.571)	**0.030**

^+^ Univariate analysis was performed using median as cutoff. *p* values in bold represent statistically significant results. Abbreviations: OS, overall survival; BMI, body mass index; BSA, body surface area; SQ, squamous cell carcinoma; LN, lymph nodes; PS, performance status; LOT, lines of treatment; PIOS, Patras Immunotherapy Score.
